# Retroperitoneal Sarcoma With Infected Necrosis: An Unfavourable Prognostic Factor

**DOI:** 10.1080/13577149877948

**Published:** 1998-12

**Authors:** Andrew J. Spillane, J. Meirion Thomas

**Affiliations:** Melanoma and Sarcoma Unit Royal Marsden Hospital Fulham Road London SW3 6JJ UK

## Abstract

*Purpose.* To report the phenomenon of infected retroperitoneal sarcoma (RPS).

*Method.* Two case reports.

*Results.* Both patients died soon after laparotomy.

*Discussion.* Infected RPS is identified as an entity not clearly documented in the literature. It should probably be added to the list of poor prognostic factors when planning the management of patients with RPS.


*Sarcoma* (1998) **2**, 179–181

ORIGINAL ARTICLE

### Retroperitoneal sarcoma with infected necrosis: an unfavourable prognostic factor

ANDREW J. SPILLANE & J. MEIRION THOMAS

*Melanoma and Sarcoma Unit, Royal Marsden Hospital, Fulham Road, London SW3 6JJ, UK*

**Abstract**

*Purpose*. To report the phenomenon of infected retroperitoneal sarcoma (RPS).
*Method*. Two case reports.
*Results*. Both patients died soon after laparotomy.
*Discussion*. Infected RPS is identified as an entity not clearly documented in the literature. It should probably be added to the list of poor prognostic factors when planning the management of patients with RPS.

**Key words**: *retroperitoneal sarcoma, infection, necrosis, prognosis.*

### Introduction

The prognosis of patients with retroperitoneal sarcoma (RPS) is poor, with an overall 5-year survival rate reported between 12–40%[1–9] with rare exception.[10] The patients with the most favourable prognosis are those with low grade tumours in which complete excision of the primary tumour is achieved.[1,2,10,11,12] Even in this group the cumulative probability of local relapse has been reported up to 85% at 5 years.[1] There is a role for palliative debulking of RPS and patients with low grade tumours can have repeated surgery over many years with a 5 year survival rate of 80%.[2] Major surgery should probably be avoided in patients with concurrent metastases, low performance status and high grade recurrent tumours unless essential for palliation.

We were involved in the management of two patients with aggressive retroperitoneal sarcomas who presented with severe pain that was unresponsive or not suitable for other palliative therapy. They were offered surgery with the hope of improving their pain. At laparotomy palliative intracapsular debulking was undertaken. Both these patients had offensive necrotic tumour and culture confirmed contamination with enteric organisms. Infected necrosis in large retroperitoneal or intraperitoneal bowel related tumours is a recognised but uncommon clinical finding[13] however there is only one report of infected RPS in the literature.[14] Infected RPS has not been related to prognosis previously.

### Case reports

### *Case 1*

This 51-year-old woman presented in January 1998 with abdominal pain and a pelvic mass. Computed tomography (CT) Scan showed a 9 cm pelvic mass thought to be arising from the uterus. At the laparotomy by her gynaecologist a retroperitoneal mass in the lower abdomen was biopsied and found to be a spindle cell sarcoma. Postoperatively she had ongoing pain that required daily morphine but was apyrexial. She was referred to the Royal Marsden Hospital and was initially advised that there was no other palliative options. There was a considerable increase in the size of the tumour on repeat CT Scan (see Fig. 1). After repeated representations by the patient and her family a further laparotomy was agreed to. There was a large pelvic mass of necrotic offensive tumour with adherent loops of small bowel. This was debulked. Postoperatively she developed sepsis and respiratory failure. It was decided not to reintubate her and she died 48 hours postoperatively. The bacterial culture from the necrotic tumour grew Streptococcus milleri, coliforms and anaerobes. Histopathology demonstrated spindle cell sarcoma (EORTC grade 2) perhaps representing gastrointestinal stromal tumour (of autonomic type).

Correspondence to: J. Meirion Thomas, Melanoma and Sarcoma Unit, Royal Marsden Hospital, Fulham Road, London SW3 6JJ, UK. Fax: 0171 351 5410; E-mail: Joseph.Thomas@rmh.nthames.nhs.uk

1357-714X/98/030179-03 $9.00 © 1998 Carfax Publishing Ltd

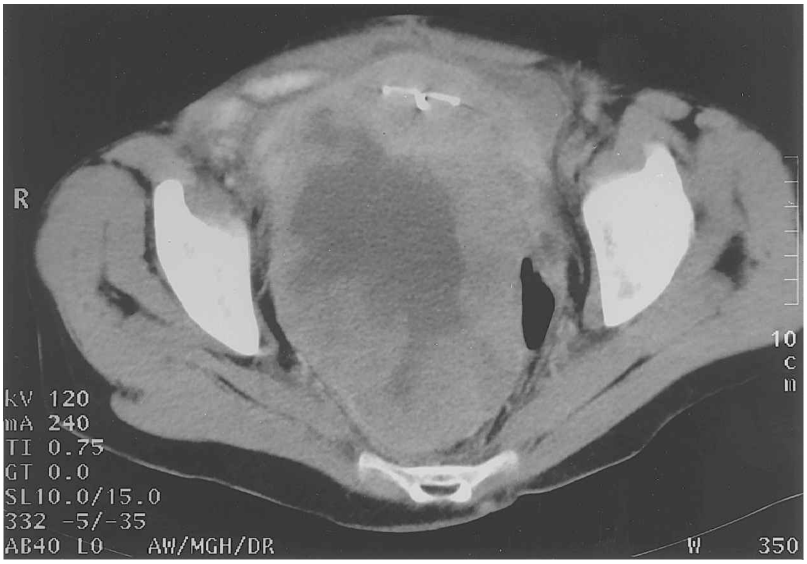

**Fig. 1.** *Large pelvic tumour with extensive necrosis. There is an intrauterine device in the uterus.*

*Case 2*

This 35-year-old woman was diagnosed with a retroperitoneal biphasic synovial sarcoma (EORTC Grade 2) in April 1997. It was incompletely resected at that time. She received 2 cycles of ifosfamide and doxorubicin which were poorly tolerated and thus stopped despite a partial response. She had several episodes of unexplained fever with the chemotherapy. In January 1998 she had progressive disease on CT Scan and poorly controlled abdominal pain. Debulking surgery was the only option to attempt to improve her quality of life. Preoperatively she had one temperature of 39°C and was commenced on broad spectrum antibiotics. At laparotomy the mass was debulked of large volumes of offensive necrotic material. Microbiological culture grew Streptococcus milleri and Bacteroides melaninogenious. Postoperatively she made good progress however she represented to hospital one month after surgery with severe abdominal pain. In the course of investigating the pain, she deteriorated and had an hypovolaemic cardiac arrest due to presumed intra-abdominal bleeding. She was initially resuscitated however under the circumstances it was considered inappropriate to intervene further. She died that evening.

### Discussion

Retroperitoneal sarcomas have a poor long term prognosis with an overall 5-year survival rate reported between 12 and 40%[1–9] with one report of 63% 5-year survival.[10] They also have an ongoing significant recurrence and mortality rate after that time.[1,5,10,11] Even when the tumour is macroscopically removed it has been reported that there is a cumulative probability of local relapse between 50–80% at 5 years.[1–10] Despite this there are many cases of recurrent low grade sarcomas that are able to be debulked at regular intervals with medium to long term survival.[2,3] The factors related to poor prognosis include metastatic disease which is often transperitoneal seeding,[1] failure to accomplish complete excision,[1–3,5,6,10–12] high grade tumours[1-3,5,9,10] and in some series the histopathological type of tumour.[3,15–17] The failure to accomplish complete excision is a manifestation of the size of the tumour. This relates to and is influenced by its location in relationship to vital structures and the number of organs involved.[1,2,4,5] To the list of poor prognostic features we would add infected RPS. The finding of infected necrosis in advanced abdominal and retroperitoneal tumours is a recognised but an uncommon clinical finding having been described in lymphoma, renal cell carcinoma and metastatic testicular carcinoma,[13] however, there is only one report of infected RPS involving a duodenal leiomyosarcoma.[14] There are no previous reports suggesting a relationship to prognosis and risks of reoperation for palliation. From our experience, if identified preoperatively infected RPS would deter further palliative surgery in favour of persisting with conservative therapy including appropriate antibiotics.

In both of our cases the nature of the aggressive disease was such that bowel was intimately involved and thus the presumed portal of entry of the infection. The bacteria cultured are known gut organisms. The other possible portal of entry is blood born organisms that seed the tumour but this is less likely given both cases were polymicrobial. No precedent exists for establishing the diagnosis of infected retroperitoneal sarcoma however there are similarities to infected necrosis in severe acute pancreatitis. Therefore CT Scan guided biopsy and culture should be feasible.[18] Similarities to infected pancreatic necrosis and its complications, such as mycotic splenic artery aneurysm causing catastrophic bleeding and multiorgan failure from sepsis, also bear striking similarity to our cases.[18]

In conclusion, retroperitoneal sarcomas have a poor prognosis. Patients should only be offered palliative surgery when their symptoms cannot be controlled by any other modality and it is technically feasible to reduce the bulk of their disease. If infected necrosis is suspected clinically CT guided aspiration for microbiological culture could be used for diagnosis. Based on our recent experience infected RPS may deter further palliative surgery in favour of conservative management. Any RPS found to have necrosis at surgery should be sent for microbiological culture as well as histopathology.

### References

1 Alvarenga J-C, Ball ABS, Fisher C, Fryatt I, Thomas JM. Limitations of surgery in the treatment of retroperitoneal sarcoma. *Br J Surg* 1991; 78:912–16.

2 Jenkins MP, Alvaranga JC, Thomas JM. The management of retroperitoneal soft tissue sarcomas. *Eur J Cancer* 1996; 32A; 4:622–26.

3 Cody HS, Turnbull AD, Fortner JG, Hadju SI. The continuing challenge of retroperitoneal sarcomas. *Cancer* 1981; 47:2147–52.

4 Adam YG, Oland J, Halevy A, Reif R. Primary retroperitoneal soft-tissue sarcoma. *J Surg Oncol* 1984; 25:8–11.

5 McGrath PC, Neifeld JP, Lawrence W *et al.*, Improved survival following complete excision of retroperitoneal sarcomas. *Ann Surg* 1984; 200:200–204.

6 Karakousis CP, Velez AF, Emrich U. Management of retroperitoneal sarcomas and patient survival. *Am J Surg* 1985; 150:376–380.

7 Salvadori B, Cusumano F, delle Donne V, de Lellis R, Conti R. Surgical treatment of 43 retroperitoneal sarcomas. *Eur J Surg Oncol* 1986; 12:29–33.

8 Solla LA, Reed K. Primary retroperitoneal sarcomas. *Am J Surg* 1986; 152:496–98.

9 Dalton RR, Donohue JH, Mucha P, van Heerden JA, Reiman HM, Chen S. Management of retroperitoneal sarcomas. *Surgery* 1989; 106:725–33.

10 Karakousis CP, Gerstenbluth R, Kontzoglou K, Driscoll D. Retroperitoneal sarcomas and their management. *Arch Surg* 1995; 130:1104–1109.

11 Heslin MJ, Lewis JJ, Nadler E, Newman E, Woodruff JM, Casper ES, Leung D, Brennan MF. Prognostic factors associated with long-term survival for retroperitoneal sarcoma: implications for management. *J Clin Oncol* 1997; 15; 8:2832–39.

12 Singer S, Corson JM, Demetri GD, Healey EA, Marcus K, Eberlein TJ. Prognostic factors predictive of survival for truncal and retroperitoneal soft-tissue sarcoma. *Ann Surg* 1995; 221; 12:185–95.

13 Kastroff KIM, Turnbull AD, Rotstein LE, Raaf JIH. Doudeno-jejunostomy and stapled occlusion for distal duodenal perforation from malignant retroperitoneal tumours. *J Surg Oncol* 1984; 26:252–55.

14 Pitcher M, Colfor A, Moskovic B, Thomas JIM. Duodenal leimyosarcoma as a cause of retroperitoneal sepsis. *Eur J Surg Oncol* 1995; 21; 1:85–86.

15 Pack GT, Tabah EJ. Primary retroperitoneal tumours: a study of 120 cases. *Surg Gynecol Obstet* 1954; 99:209–231, 313–41.

16 Jacobsen S, Juul-Jorgensen S. Primary retroperitoneal tumours. *Acta Chir Scand* 1974; 140:498–500.

17 Stower MJ, Hardcastle ID. Malignant retroperitoneal sarcoma; a review of 32 cases. *Clin Oncol* 1982; 8:257–63.

18 Rattner DW, Warshaw AL. Acute panreatitis. In: Morris PJ and Malt RA, eds *Oxford Textbook of Surgery*. Oxford: Oxford University Press, 1994:1289–98.
